# Evasion of Host Defence by *Leishmania donovani*: Subversion of Signaling Pathways

**DOI:** 10.4061/2011/343961

**Published:** 2011-04-27

**Authors:** Md. Shadab, Nahid Ali

**Affiliations:** Infectious Diseases and Immunology Division, Indian Institute of Chemical Biology, 4, Raja S.C. Mullick Road, Kolkata 700032, India

## Abstract

Protozoan parasites of the genus *Leishmania* are responsible for causing a variety of human diseases known as leishmaniasis, which range from self-healing skin lesions to severe infection of visceral organs that are often fatal if left untreated. *Leishmania donovani* (*L. donovani*), the causative agent of visceral leishmaniasis, exemplifys a devious organism that has developed the ability to invade and replicate within host macrophage. In fact, the parasite has evolved strategies to interfere with a broad range of signaling processes in macrophage that includes Protein Kinase C, the JAK2/STAT1 cascade, and the MAP Kinase pathway. This paper focuses on how *L. donovani* modulates these signaling pathways that favour its survival and persistence in host cells.

## 1. Introduction

Leishmaniasis, caused by more than 20 species of *Leishmania* and transmitted by approximately 30 species of sand fly, is one of the major infectious diseases affecting 12 million people worldwide [1–3]. *Leishmania* are obligate intracellular parasites that infect the hematopoietic cells of the monocyte/macrophage lineage. They exhibit dimorphic life cycle, residing as extracellular flagellate promastigotes in the digestive tract of the sand fly vector and as intracellular flagellate amastigotes within macrophage phagolysosome in their mammalian host [4]. Depending upon the type of species, infection results in a spectrum of clinical manifestations ranging from self-healing skin ulcers to disfiguring mucosal lesions to life-threatening infections of visceral organs (liver and spleen). Among all these forms, visceral leishmaniasis (VL, also known as kala-azar), caused by *Leishmania donovani* complex (i.e., *L. donovani *and *L. infantum *in Old World and *L. chagasi *in New World), is often fatal if left untreated. An estimated annual incidence of 0.5 million new cases of VL is reported to occur in 62 countries [5].

 Monocytes and macrophages are considered as sentinels of the immune system. These cells participate in innate immunity and act as the first line of defence in immune response to foreign invaders. They also participate in initiation of the acquired immune response by ingesting foreign particles and presenting them on their surface with major histocompatibility (MHC) complex. In their resting stage, macrophages are relatively quiescent, showing low levels of oxygen consumption, MHC class II gene expression, and cytokine secretion. But once activated, they exhibit maximal secretion of factors like IL-1, IL-6, TNF-*α*, reactive oxygen species, and nitric oxide produced by inducible nitric oxide synthase (iNOS) [6]. Production of reactive nitrogen and oxygen intermediates (RNIs and ROIs) has made these cells potentially microbicidal [7, 8]. In spite of these, a pathogen like *L. donovani* is able to replicate and survive inside these cells. This suggests that this pathogen has evolved intricate mechanisms to evade or impair macrophage antimicrobial functions [9].

The parasite has been observed to interfere with the host signal transduction in a way that the effector function of macrophage gets impaired, which in turn facilitates parasite survival. Signalling pathways inside the cell are tightly regulated by protein phosphorylation, and levels of cellular protein phosphorylation are controlled by the activities of both protein kinases and protein phosphatases [10, 11]. Therefore, it is not surprising that the parasite interferes with the protein phosphorylation process, impairing kinase-phosphatase balance, and hence distorting macrophage's antimicrobial functions. This paper, therefore, highlights the molecular mechanism by which *L. donovani* modulates macrophage's signalling machinery that promotes its intracellular survival and propagation within the host.

## 2. MAPK Mediated Pathway

Mitogen-activated protein kinases (MAPKs), a group of serine/threonine-specific protein kinases, constitute one of the important intracellular signalling pathways in eukaryotic cells like macrophages, regulating their accessory and effector functions including production of proinflammatory cytokines and NO [12]. MAPK family includes extracellular signal-related kinases 1 and 2 (ERK1/2), c-jun NH_2_-terminal kinase (JNK), and p38 MAPK. Activation of these kinases requires dual phosphorylation of serine/threonine and tyrosine residues, located in a Thr-X-Tyr motif in their regulatory domain [12, 13], by upstream kinases like MAP/ERK Kinase (MEK), which is itself activated by MEK Kinase (MEKK) [7]. Once activated, these kinases phosphorylate a number of selected intracellular proteins including the ubiquitous transcription factors such as activating protein 1 (AP-1), NF-*κ*B and IFN regulatory factors (IRFs), because of which a diverse signalling cascade is triggered that regulates gene expression by transcriptional and posttranscriptional mechanisms [14–16]. A number of studies implicated that *L. donovani* infection of macrophage leads to the alteration of MAP Kinase pathway, which in turn promotes parasite survival and propagation within the host cell. For example, Phorbol 12-myristate 13-acetate- (PMA-) dependent activation of MAP kinase and subsequent expression of c-Fos and elk-1 is impaired in macrophage infected with *L. donovani* [17]. Further, the observation that these effects largely negate when the macrophage is treated with sodium orthovanadate prior to infection [17] suggests that* Leishmania-*induced cellular phosphotyrosine phosphatases are responsible for resulting in such macrophage deactivation. In fact, it was found that the specific activity of Src homology 2 (SH2) domain containing PTP (SHP-1) towards MAP Kinase increases in *L. donovani-*infected macrophage. Consistent to this, there was also reported an increased activity of SHP-1 as well as that of total PTP [18], which apparently supports the finding that SHP-1-deficient macrophage unlike normal macrophage activates JAK2, ERK1/2, and the downstream transcription factors, NF-*κ*B and AP-1 and NO production when treated with IFN-*γ* even in infected conditions [19]. Recently, Kar et al. demonstrated some different phosphatases that are also induced during *Leishmania* infection and promotes parasitic survival. These specific MAPK-directed phosphatases, MKP1 and PP2A, are shown to inhibit ERK1/2 MAP Kinase resulting in diminished expression of iNOS mRNA [20].

**Figure 1:**
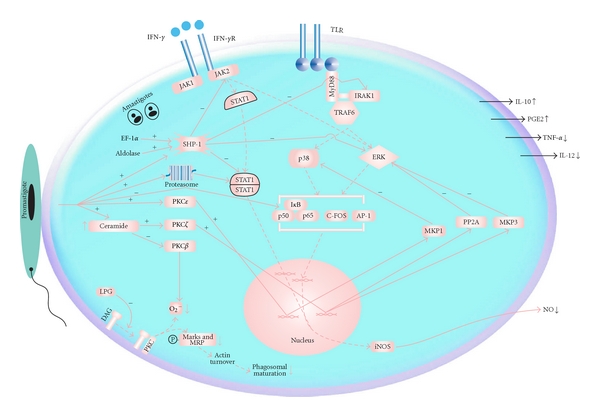
Manipulation of macrophage signaling pathways by *L. donovani. L. donovani-*derived molecules like EF-1*α* and Fructose 1,6 bisphosphate aldolase activate SHP-1 which negatively affects JAK2, STAT1, ERK1/2, and IRAK-1 inhibiting IFN-*γ*-induced NO production and TLR-mediated production of cytokines like IL-12, TNF-*α*. Impairment of IFN-*γ*-dependent pathway also includes reduced level of JAK2 expression and proteasome-mediated degradation of STAT1*α*. NF-*κ*B-dependent pathway is blocked by impaired degradation of I*κ*B. Other than SHP-1, *L. donovani *also leads to an enhanced expression of MKP3, PP2A, and MKP1 by inducing PKC*ζ*, and PKC*ε* respectively. These dual phosphatases or serine/threonine phsophatases inhibit p38 (MKP1) and ERK1/2 (MKP3/PP2A) resulting in upregulation of IL-10 and downregulation of NO and TNF-*α* production. PKC-mediated secretion of immunosuppressive molecule PGE2 is also observed. Enhanced level of endogenous ceramide inhibits PKC*β* leading to an impaired oxidative burst. PKC-dependent phosphorylation of MARKS and MRP and resulting phagosomal maturation is also inhibited by the parasite. Solid lines: Interaction or positive/negative modulation; dashed lines, interrupted pathway; MyD88: myeloid differentiation primary-response gene 88; IRAK1: IL-1R-associated Kinase 1; TRAF6: TNF receptor-associated factor 6.

Additional possible mechanism of MAP Kinase inactivation by *Leishmania* could be explained by the elevation of endogenous ceramide in parasitised macrophage [21]. Ceramide is an intracellular lipid mediator, which plays an important role in regulating such diverse responses as cell cycle arrest, apoptosis, and cell senescence [22]. It exerts its cellular functions by means of a delicate regulation of downstream kinases and phosphatases [23]. It was found that intracellular ceramide dephosphorylates ERK by activating tyrosine phosphatase [21]. This impairment of ERK is further shown to attenuate AP-1 and NF-*κ*B transactivation and production of NO in infected macrophage [24] (Figure 1). Moreover, these results are in agreement with previous reports that infection of naïve macrophage with promastigotes of *L. donovani* evades activation of MAPKs leading to impaired proinflammatory cytokines production [25]. However, treatment of macrophage with IFN-*γ* prior to infection is also shown to induce the phosphorylation of p38 MAPK and ERK1/2 and production of proinflammatory cytokines [25]. Recently, it was identified that priming of macrophage with IFN-*γ* lead to the expression of Toll-like Receptor 3 (TLR3) which is recognised by the parasite, leading to production of proinflammatory cytokines like tumor necrosis factor alpha (TNF-*α*) and NO [26]. TLR-mediated regulation of MAP Kinase in macrophage infected with* Leishmania *was also demonstrated by Chandra and Naik. They showed that *L. donovani* infection of both THP-1 cells and human monocytes downregulates Toll-like Receptor 2 (TLR2) and Toll-like Receptor 4 (TLR4), stimulated IL-12p40 production and increases IL-10 production, by suppressing MAPK P38 phosphorylation and activating ERK1/2 MAP kinase phosphorylation through a contact-dependent mechanism [27]. As previous studies have shown that TLR ligation results in phosphorylation of MAPK p38 and ERK1/2 leading to the production of IL-12 and IL-10, respectively [28, 29]. Therefore, it seems that *Leishmania* infection modulates macrophage function by counter regulating p38 and ERK1/2 phosphorylation. In addition, such differential regulation is a direct implication of parasitic infection without much influence of cytokines and is evidenced by the observation that neutralisation of macrophage with anti-IL-10 antibody prior to infection did not abrogate the suppression of IL-12 production [27]. A recent study by Rub et al. [30] also showed that *L. major* infection of macrophage inhibits CD40-induced phosphorylation of the kinases Lyn and p38 resulting in diminished production of IL-12, whereas it upregulates CD40-induced phosphorylation of the Kinases Syk and ERK1/2 and enhances the production of IL-10. Moreover, this has been found to be dependent on the assembly of distinct CD40 signalosomes which is influenced by the level of membrane cholesterol. This represents a unique strategy that the parasite has evolved to survive and needs to be investigated in case of *L. donovani* infection of host macrophage. 

The synthetic* Leishmania* molecule LPG is also shown to exhibit differential regulation of MAP kinase pathway in J774 macrophage [31]. By stimulating ERK MAP kinase that inhibits macrophage IL-12 production, LPG has been shown to skew the immune response towards Th 2 type. This suggests that *Leishmania* parasite employs this molecule to evade macrophage effector function [31]. But a subsequent study by Privé and Descoteaux contradicted these results that LPG instead of stimulating ERK exerts inhibitory effect on ERK activation in murine bone marrow-derived macrophage (BMM) [25]. In addition, the influence of LPG on ERK is specific as infection of naïve macrophage with LPG defective parasite was shown to induce ERK activation while having insignificant effect on p38 and JNK MAP kinase activation [25]. These findings are further supported by a the observation that inhibition of the ERK pathway with PD059089 (ERK inhibitor) increases the parasitic survival in infected macrophage either through increased uptake or decreased killing of the parasite [32]. Nevertheless, recently it has also been explained that the *Leishmania *surface molecule LPG stimulates the simultaneous activation of all three classes of MAP kinases, ERKs, JNK, and the p38 MAP kinase with differential kinetics in J774A.1 macrophage with production of IL-12 and NO [33]. In conclusion, these demonstrations suggest that use of different macrophages in respective studies might have contributed to these contradictory results and therefore needs additional studies to address such disparities in alteration of signal transduction pathways in response to *Leishmania *infection.

Several studies on *Leishmania*-dependent modulation of MAP Kinase pathway implicates that regulation of p38 activation in host macrophage is important in the control of *Leishmania* infection. For example, it was demonstrated that treatment of macrophage with anisomycin, which activates p38, diminishes the survival of the parasite in macrophage [32]. This is in consistency with a current finding that testosterone suppresses* L. donovani*-induced activation of p38 and enhances the persistence of the parasite in macrophage [34]. Furthermore, the observation that the specific MAPK-directed phosphatase, MKP1, induced by *L. donovani* infection downregulated p38 activation and enhanced the survival of the parasite in macrophage again emphasizes the importance of p38 MAP Kinase activation in *Leishmania *infection [20].

## 3. Protein Kinase C-Dependent Pathway

Protein kinase C (PKC) is a family of calcium and phospholipid-dependent serine/threonine kinases having closely related structures. Based on their intracellular distribution, cofactor requirement, and substrate specificities, these have been grouped into three subfamilies, namely, classical PKCs (cPKC; *α*, *β*, *γ*), novel PKCs (nPKC; *δ*, *ε*, *η*, *θ*), and atypical PKCs (aPKCs; *ζ*, *ι*, *λ*), [35–37]. While classical PKCs are activated by the intracellular second messengers Ca^2+^ and diacylglycerol (DAG) together with the membrane lipid phosphatidylserine (PS), novel PKCs are activated by diacylglycerol and phosphatidylserine; and atypical PKCs, whose activity is yet not clearly determined, are apparently shown to be stimulated by phosphatidylserine [38]. These kinases reside in the cytosol of the cell in their inactive conformation. Upon activation by stimuli like hormones or phorbol esters, they translocate to cell membrane or to different cell organelles. The mechanism of activation and the localization to subcellular compartments varies among the various isoforms [38]. PMA, a well-known phorbol ester, has been shown to activate [39] and deplete [40] PKC from cells depending upon the time of incubation. *L. donovani* has been shown to evade several macrophage microbicidal activities by altering PKC-mediated signaling pathways. 

*L. donovani* promastigotes, amastigotes, and its major surface molecule LPG have been shown to inhibit PKC-mediated c-fos gene expression in murine macrophage while exhibiting little or no effect on PKA-mediated gene expression [41, 42]. This suggests that the parasite has selectively evolved PKC inhibitory mechanisms, which assist in its survival and propagation within the host macrophage. Interestingly, the observation that LPG-deficient amastigotes are also able to inhibit PKC-mediated c-fos gene expression [41], and PKC activity [43] implicates the role of additional *Leishmania* molecules in blocking PKC-mediated events. Indeed, McNeely et al. demonstrated GIPL to be responsible for PKC inactivation in vitro [44], although its role in intact macrophage still needs to be determined. *L. donovani* infection of human monocytes has also been shown to attenuate PMA-induced oxidative burst activity and protein phosphorylation, by impairing PKC activation [43]. Phosphorylation of both the PKC-specific VRKRTRLLR substrate peptide and MARCKS and endogenous PKC substrate is also shown to be inhibited by LPG treatment of macrophage [45]. Giorgione et al. further demonstrated by an assay using large unilamellar vesicles that LPG inhibits PKC-*α* catalyzed phosphorylation of histone proteins. This study also showed that inhibition is likely a result of alterations in the physical properties of the membrane [46] and supports a recent finding that uptake and multiplication of parasite increases in PKC-depleted macrophage having diminished membrane microviscosity [47]. The level of MARCKS-related proteins (MRP, MacMARCKS) in macrophage is found to be attenuated by all species and strains of* Leishmania* parasites, including LPG-deficient *Leishmania major* L119 [48]. Thus, this indicates that *Leishmania* parasites, in addition of impairing PKC-dependent protein phosphorylation, have developed a novel mechanism to modulate downstream PKC substrates, which interferes with PKC-mediated signalling pathways. Furthermore, the observation that depletion of PKC renders macrophages more permissive to the proliferation of *L. donovani* again reinforces the fact that inhibition of PKC-dependent events is one of the important strategies that the parasite employs, for promoting its survival within the host cell [45]. 

One of the studies demonstrated that mere attachment of parasite on macrophage surface leads to the activation of PKC and production of O_2_^−^ and NO, whereas internalization of the parasite inhibits these responses [49]. From such observations, it was suggested that *L. donovani* attached to the surface of host cell during initial phase of infection behaves like other organisms that are killed by macrophages. But once they are internalized, triggering of these effector molecules like O_2_^−^ and NO is switched off in part, due to the impairment of PKC-mediated signal transduction pathways. 

The finding that the activity of PKC increases after it attaches to the plasma membrane in infected macrophage appears to indicate that translocation of PKC isoforms remains unaffected during *Leishmania* infection [49, 50]. However, the affinity of these isoforms towards their activator DAG is shown to be diminished in infected macrophage correlating reduced generation of oxygen radicals [43]. Furthermore, this reduction in affinity has been suggested to be linked with direct interference of LPG in binding of the regulators like calcium and DAG to PKC [45]. LPG is also shown to inhibit phagosomal maturation, a process requiring depolymerization of periphagosomal F-actin [51]. Holm et al. demonstrated that treatment of macrophage with LPG induces the accumulation of periphagosomal F-actin, which was found to be associated with impaired recruitment of the lysosomal marker LAMP1 and PKC*α* to the phagosome [52]. Recently, it was demonstrated that PKC-*α* is involved in F-actin turnover in macrophages and PKC-*α*-dependent breakdown of periphagosomal F-actin is required for phagosomal maturation [53]. Therefore, there is no doubt that LPG inhibits phagosomal maturation by impairing PKC-*α*-dependent depolymeristion of F-actin, resulting in enhanced intracellular survival of the parasite in infected macrophage [53]. These findings further corroborate the previous observations that intracellular survival of the parasite was enhanced by 10- to 20-fold in the murine macrophage cell line RAW 264.7 overexpressing a dominant-negative (DN) mutant of PKC-*α* [54].

Infection of murine cells in vivo and in vitro with *Leishmania* parasite has been shown to induce an increased synthesis of prostaglandin E2 (PGE2) that favours parasite persistence and progression [55, 56]. Recently, it was demonstrated that generation of PGE2 in *L. donovani*-infected U937 human monocytes is, in part, dependent upon PKC-mediated signalling pathway [57]. This shows that *L. donovani*, in addition to downmodulating macrophage functions by affecting important signalling pathways, induces secretion of immunosuppressive molecules (e.g., PGE2) to potentially affect functions of surrounding uninfected cells, which in turn renders macrophage suitable for the survival and establishment of the parasite.

*L. donovani* infection of macrophage, whereas selectively attenuates both the expression and activity of calcium-dependent PKC-*β*, is shown to induce the expression and activity of calcium-independent PKC-*ζ* isoform with diminished production of O_2_^−^ and TNF-*α* [58]. Attenuation of the expression and activity of calcium-dependent PKC-*β* has been suggested to be mediated by IL-10 overproduction, as pretreatment of infected macrophage with neutralizing anti-IL-10 restoring the activity of PKC as well as production of O_2_^−^, NO, and TNF-*α* [59]. From these findings, it can be thus speculated that *L. donovani* infection induces endogenous secretion of murine IL-10, in order to facilitate its intracellular survival via selective impairment of PKC-mediated signal transduction. One possible mechanism for this differential regulation of both the expression and activity of PKC isotypes by *Leishmania *infection was demonstrated by Ghosh et al. They elaborated that *Leishmania *infection induces elevation of intracellular ceramide in infected macrophage largely due to its denovo synthesis. The enhanced ceramide then downregulates classical calcium-dependent PKC, enhances expression of atypical PKC-*ζ* isoform, and diminishes MAPK activity and generation of NO [21]. Consistent with this, Dey et al. also reported ceramide-mediated upregulation of atypical PKC-*ζ* isoform in infected macrophage. However, they further showed that this ceramide-induced atypical PKC-*ζ* inhibits PKB (Akt) phosphorylation which is dependent upon PKC*ζ*-Akt interaction, as the treatment of the cell with PKC-*ζ* inhibitor prior to infection showed a significant translocation of Akt from cytoplasm to the membrane [60]. Moreover, *L. donovani *infection of macrophage has been found to induce the expression of MAPK-directed phosphatases such as MKP1, MKP3, and a thronine/serine phosphatase PP2A by stimulating various PKC isoforms. While MKP3 and PP2A, activated by PKC-*ζ* were further found to be responsible for ERK1/2 dephosphorylation, MKP1 induced by PKC-*ε* is shown to inhibit p38 phosphorylation, which resulted in diminished production of NO and TNF alpha favouring enhanced survival of the parasite in macrophage [20]. In conclusion, the observation that C-C chemokines restore calcium-dependent PKC activity and inhibit calcium-independent atypical PKC activity in *L. donovani*-infected macrophages under both in vivo and in vitro conditions restricting the parasitic load again supports the fact that impairment of PKC-mediated signaling is a key to the establishment of *Leishmania* parasites in their host cells [61].

## 4. JAK2/STAT1-Dependent Pathway

IFN-*γ* is a potent cytokine that induces macrophage activation and helps resisting *Leishmania* infection [62, 63]. It mediates its biological functions via IFN-*γ* receptor- (IFN-*γ*R-) mediated pathway involving receptor-associated kinases JAK1/JAK2 and STAT-1 [64, 65]. Binding of IFN-*γ* to its multisubunit receptor triggers its dimerization and allows transphosphorylation of the Jak1 and Jak2. These kinases in turn phosphorylate the cytoplasmic tail of the receptor itself which recruits the cytoplasmic molecule STAT1*α*. This transcription factor is then phosphorylated, becomes a homodimer, and then translocates to the nucleus to enhance transcription of IFN-*γ*-induced genes, such as FcgRI [66]. *Leishmania* induced macrophage dysfunction such as defective production of NO [67] and MHC [68] expression in response to IFN-*γ* may not exclude the possibility that the parasite could have impaired this pathway. In fact, a number of studies implicated *Leishmania*-mediated impairment of JAK2/STAT1 pathway, which correlates with such macrophage deactivation. For instance, one of the studies showed that *L. donovani* infection attenuates IFN-*γ*-induced tyrosine phosphorylation and selectively impairs IFN-*γ*-induced Jak1 and Jak2 activation and phosphorylation of Stat1 in both differentiated U-937 cells and human monocytes [69]. A probable mechanism for this was demonstrated by Blanchette et al. that *L. donovani *infection of macrophage rapidly induces host PTP activity simultaneously with dephosphorylation of macrophage protein tyrosyl residues and inhibition of protein tyrosine kinase [18]. They further revealed that upon infection, PTP SHP-1 is also rapidly induced, which interacts strongly with JAK2, and impairs IFN-*γ* signaling [18]. However, a recent observation that IFN-*γ*-stimulated STAT1*α* activity is also reduced in SHP-1-deficient macrophages following *L. donovani* infection indicates that *Leishmania* employs further mechanisms to inhibit STAT1 activity [19]. One possible mechanism could be the proteasome-mediated degradation of STAT1*α* in infected macrophage, as treatment of macrophage with proteasome inhibitors prior to infection is shown to rescue STAT1*α* nuclear translocation as well as restore its general protein level in *Leishmania*-infected macrophage [70]. Additionally, *L. donovani* infection of macrophage has been shown to attenuate IFN-*γ*R alpha subunit expression [71] and induce the transient expression of the cytokine signaling 3 (SOCS3) [72], which also shown to negatively regulate IFN-*γ* signaling. More recently, *L. donovani* amastigote is found to inhibit the expression of IRF-1 while having no effect on STAT1*α* protein levels. This inhibition of IRF-1 expression correlates with the defective nuclear translocation of STAT1 and further revealed that the IFN-induced STAT1*α* association with the nuclear transport adaptor importin-5 is compromised in *L. donovani *amastigote-infected macrophage [73]. These results thus provide evidence for a novel mechanism used by *L. donovani* to interfere with IFN-*γ*-activated macrophage functions.

## 5. Implication of Phosphatases

Protein phosphatases are key regulatory components in signal transduction pathways [74, 75]. Based on their substrate specificity, these have been divided into two main groups. One of them specifically hydrolyzes serine/threonine phosphoesters (PPs) and the other is phosphotyrosine specific called protein tyrosine phosphatases (PTPs). Apart from these, a subfamily of PTPs also exists that are capable of efficiently hydrolysing both phosphotyrosine and phosphoserine/threonine residues and are therefore known as dual-specificity phosphatases. PTP-regulated protein dephosphorylation is a critical control mechanism for numerous physiological processes such as cell growth, motility, metabolism, cell cycle regulation, and cytoskeletal integrity [76, 77]. However, for parasites like *Leishmania* these molecules have been proved fruitful in enhancing their survival within host macrophage by inhibiting several intracellular signaling cascades involved in host effector functions. 

### 5.1. SHP-1 Protein Tyrosine Phosphatase

Protein Tyrosine Phosphatases containing Src homology 2 (SH2) domains have been identified in a wide variety of species [74, 75]. One of them is PTP SHP-1, which is also known as PTP1C, HCP, SHPTP1, and SHP [75]. This phosphatase is expressed not only in haematopoietic cells but also in smooth muscle [78] and epithelial cells [79] and is considered as an important negative regulator of numerous signaling pathways, such as those related to the actions of interferons [80, 81] and erythropoietin [82, 83].

Structural analysis of SHP-1 showed that this phosphatase contains two SH2 domains in its N-terminal portion, a phosphatase domain conserved in a central position and a C-terminal tail [84]. The SH2 domains which contain specific amino acid sequences have been found to interact with the target protein through an immunoreceptor tyrosine-based inhibitory motif (ITIMs) within the consensus sequence I/V/LxYxxL/V [85]. These specialized motifs are known to be present in many signaling molecules [86, 87], and multiple types of ITIMs exist and display specific abilities to recruit and activate SH2 containing PTPs. SHP-1 has been shown to bind to receptors and dephosphorylate them directly or associate with a receptor and dephosphorylate other members of the receptor binding complex. Moreover, it also interacts with other cytosolic proteins and was found to dephosphorylate them or their associated proteins [86]. Several studies on *Leishmania* infection have implicated a negative role of these phosphatases. 

A study by Olivier et al. for the first time demonstrated a role of protein tyrosine phosphatases in *Leishmania* infection, by using PTP inhibitors such as the peroxovanadium (pv) compound bpv(phen), which restricted the progression of both visceral and cutaneous leishmaniasis in vitro as well as in vivo [88]. Consistent with this, Blanchette et al. showed that *L. donovani *infection of macrophage induces a rapid elevation of total PTP activity and SHP-1 activity, leading to a widespread dephosphorylation of high-molecular-weight proteins [18]. In addition, activated SHP-1 is observed to interact with JAK2 and impair its activation in response to IFN-*γ* [18]. Accordingly, it was also found that *Leishmania*-induced SHP-1 interacts strongly with MAP kinases and impairs PMA-stimulated ERK1/2 phosphorylation, Elk-1 activation, and c-fos mRNA expression resulting in attenuated expression of iNOS [17]. These results are strongly supported by a recent finding that infection of SHP-1 deficient macrophage with *L. donovani* exhibits normal JAK2 and ERK1/2 activity and increased NO production in response to IFN*γ* [19]. Taken together, these findings suggest that *L. donovani* exploits host PTP SHP-1 in modulating several key signalling molecules to evade macrophage effector functions.

Studies aimed at understanding the mechanism responsible for the change in activation state of SHP-1 led to the identification of *Leishmania* EF-1*α* and subsequently fructose-1,6 bisphosphate aldolase, which were shown to bind and activate PTP SHP-1 in vitro and in vivo, in a similar fashion [89, 90]. In both these cases, although the trafficking mechanism of the molecules is not yet clear, it appears that they are exported out of the phagosome into the cytosol, where they activate SHP-1 [89, 90]. These observations lead to the speculation that more than one *Leishmania*-derived molecule is likely to be needed for optimum activation of SHP-1 as these molecules are reported to cooperate in activating this PTP by interacting at different sites on it [90]. 

SHP-1 is also shown to inhibit a critical kinase (IRAK-1) involved TLR signaling. This has been linked to a rapid binding of SHP-1 with IRAK-1 through an evolutionarily conserved ITIM-like motif identified in the kinase. This motif was also present in other kinases involved in Toll signalling and therefore could represent a regulatory mechanism of relevance to many kinases. This work therefore reports a unique mechanism by which *Leishmania* can avoid harmful TLR signalling [91].

### 5.2. Other Phosphatases

It is apparent from several studies that SHP-1 plays an important role in pathogenesis during *Leishmania* infection. Nevertheless, the finding that SHP-1-deficient macrophage witnessed an increased PTP activity and inhibition of NF-*κ*B and AP-1 during *L. donovani* infection points to the induction of additional PTPs that could also be involved in disease progression [19]. In fact, Olivier et al. showed that macrophage PTP-1B is rapidly induced upon *Leishmania* infection (Gomez and Olivier, unpublished data), although the underlying mechanism involved in its activation and in its enrolment in macrophage dysfunction during* L. donovani* infection remains undiscovered and needs further investigations. The elevated level of endogenous ceramide, generated during *Leishmania* infection, is shown to activate a vanadate-sensitive tyrosine phosphatase which dephosphorylates ERK1/2 resulting in a diminished production of NO [21]. Similarly, Dey et al. described another phosphatase PP2A, induced during *L. donovani* infection of macrophage, mediated through ceramide. PP2A was found to inhibit PKB (Akt), a kinase involved in respiratory burst activity in infected macrophage, and enhanced survival of the parasite in infected macrophage [60]. *L. donovani* infection of macrophage is also shown to induce a significant upregulation of a serine/threonine phosphatase PP2A and two specific MAPK-directed phosphatases such as MKP1 and MKP3. [20]. While MKP3- and PP2A- mediated dephosphorylation of ERK1/2 resulted in substantial decrease in iNOS expression in infected macrophage, MKP1 is shown to skew cytokine balance towards Th2 response that favoured persistence and propagation of the disease in in vitro as well as in vivo model of *Leishmania* infection [20].

## 6. Conclusion

Parasitic protozoa like *Leishmania *are a major cause of severe morbidity and mortality in several parts of the world. These pathogens have evolved with the mammalian immune system and typically produce long lasting chronic infections. They exhibit an efficient survival in host macrophage by manipulating host signaling machinery in its favour. This paper has covered some of these mechanisms which would facilitate further studies in knowing the unidentified strategies that the parasite employs in subverting host immune system. Moreover, given that these signalling pathways could be manipulated pharmacologically, an improved understanding of the host parasite interaction would allow the development of new therapies to control such infectious agents.

## References

[1] Pearson RD, De Queiroz Sousa A (1996). Clinical spectrum of leishmaniasis. *Clinical Infectious Diseases*.

[2] Killick-Kendrick R (1990). Phlebotomine vectors of the leishmaniases: a review. *Medical and Veterinary Entomology*.

[3] World Health Organisation The leishmaniasis and Leishmania/HIV co-infections. http://www.who.int/mediacentre/factsheets/fs116/en/print.html.

[4] Liew FY, O’Donnell CA (1993). Immunology of Leishmaniasis. *Advances in Parasitology*.

[5] Sundar S, Chatterjee M (2006). Visceral leishmaniasis—current therapeutic modalities. *Indian Journal of Medical Research*.

[6] Fearon DT, Locksley RM (1996). The instructive role of innate immunity in the acquired immune response. *Science*.

[7] MacMicking J, Xie QW, Nathan C (1997). Nitric oxide and macrophage function. *Annual Review of Immunology*.

[8] Vazquez-Torres A, Fang FC (2001). Oxygen-dependent anti-Salmonella activity of macrophages. *Trends in Microbiology*.

[9] Olivier M, Gregory DJ, Forget G (2005). Subversion mechanisms by which Leishmania parasites can escape the host immune response: a signaling point of view. *Clinical Microbiology Reviews*.

[10] Su B, Karin M (1996). Mitogen-activated protein kinase cascades and regulation of gene expression. *Current Opinion in Immunology*.

[11] Hunter T (1995). Protein kinases and phosphatases: the yin and yang of protein phosphorylation and signaling. *Cell*.

[12] Seger R, Krebs EG (1995). The MAPK signaling cascade. *FASEB Journal*.

[13] Cobb MH (1999). MAP kinase pathways. *Progress in Biophysics and Molecular Biology*.

[14] Karin M (1996). The regulation of AP-1 activity by mitogen-activated protein kinases. *Philosophical Transactions of the Royal Society B*.

[15] Kamijo R, Harada H, Matsuyama T (1994). Requirement for transcription factor IRF-1 in NO synthase induction in macrophages. *Science*.

[16] Murphy TL, Cleveland MG, Kulesza P, Magram J, Murphy KM (1995). Regulation of interleukin 12 p40 expression through an NF-*κ*B half-site. *Molecular and Cellular Biology*.

[17] Nandan D, Lo R, Reiner NE (1999). Activation of phosphotyrosine phosphatase activity attenuates mitogen- activated protein kinase signaling and inhibits c-FOS and nitric oxide synthase expression in macrophages infected with *Leishmania donovani*. *Infection and Immunity*.

[18] Blanchette J, Racette N, Faure R, Siminovitch KA, Olivier M (1999). Leishmania-induced increases in activation of macrophage SHP-1 tyrosine phosphatase are associated with impaired IFN-*γ*-triggered JAK2 activation. *European Journal of Immunology*.

[19] Forget G, Gregory DJ, Whitcombe LA, Olivier M (2006). Role of host protein tyrosine phosphatase SHP-1 in *Leishmania donovani*-induced inhibition of nitric oxide production. *Infection and Immunity*.

[20] Kar S, Ukil A, Sharma G, Das PK (2010). MAPK-directed phosphatases preferentially regulate pro- and anti-inflammatory cytokines in experimental visceral leishmaniasis: involvement of distinct protein kinase C isoforms. *Journal of Leukocyte Biology*.

[21] Ghosh S, Bhattacharyya S, Das S (2001). Generation of ceramide in murine macrophages infected with *Leishmania donovani* alters macrophage signaling events and aids intracellular parasitic survival. *Molecular and Cellular Biochemistry*.

[22] Hannun YA (1996). Functions of ceramide in coordinating cellular responses to stress. *Science*.

[23] Dobrowsky RT, Kamibayashi C, Mumby MC, Hannun YA (1993). Ceramide activates heterotrimeric protein phosphatase 2A. *Journal of Biological Chemistry*.

[24] Ghosh S, Bhattacharyya S, Sirkar M (2002). *Leishmania donovani* suppresses activated protein 1 and NF-*κ*B activation in host macrophages via ceramide generation: involvement of extracellular signal-regulated kinase. *Infection and Immunity*.

[25] Privé C, Descoteaux A (2000). *Leishmania donovani* promastigotes evade the activation of mitogen-activated protein kinases p38, c-Jun N-terminal kinase, and extracellular signal-regulated kinase-1/2 during infection of naive macrophages. *European Journal of Immunology*.

[26] Flandin JF, Chano F, Descoteaux A (2006). RNA interference reveals a role for TLR2 and TLR3 in the recognition of *Leishmania donovani* promastigotes by interferon-*γ*-primed macrophages. *European Journal of Immunology*.

[27] Chandra D, Naik S (2008). *Leishmania donovani* infection down-regulates TLR2-stimulated IL-12p40 and activates IL-10 in cells of macrophage/monocytic lineage by modulating MAPK pathways through a contact-dependent mechanism. *Clinical and Experimental Immunology*.

[28] Suttles J, Milhorn DM, Miller RW, Poe JC, Wahl LM, Stout RD (1999). CD40 signaling of monocyte inflammatory cytokine synthesis through an ERK1/2-dependent pathway: a target of interleukin (IL)-4 and IL-10 anti- inflammatory action. *Journal of Biological Chemistry*.

[29] Lu HT, Yang DD, Wysk M (1999). Defective IL-12 production in mitogen-activated protein (MAP) kinase kinase 3 (Mkk3)-deficient mice. *EMBO Journal*.

[30] Rub A, Dey R, Jadhav M (2009). Cholesterol depletion associated with *Leishmania major* infection alters macrophage CD40 signalosome composition and effector function. *Nature Immunology*.

[31] Feng GJ, Goodridge HS, Harnett MM (1999). Extracellular signal-related kinase (ERK) and p38 mitogen-activated protein (MAP) kinases differentially regulate the lipopolysaccharide-mediated induction of inducible nitric oxide synthase and IL-12 in macrophages: *Leishmania* phosphoglycans subvert macrophage IL-12 production by targeting ERK MAP kinase. *Journal of Immunology*.

[32] Junghae M, Raynes JG (2002). Activation of p38 mitogen-activated protein kinase attenuates *Leishmania donovani* infection in macrophages. *Infection and Immunity*.

[33] Balaraman S, Singh VK, Tewary P, Madhubala R (2005). *Leishmania* lipophosphoglycan activates the transcription factor activating protein 1 in J774A.1 macrophages through the extracellular signal-related kinase (ERK) and p38 mitogen-activated protein kinase. *Molecular and Biochemical Parasitology*.

[34] Liu L, Wang L, Zhao Y, Wang Y, Wang Z, Qiao Z (2006). Testosterone attenuates p38 MAPK pathway during *Leishmania donovani* infection of macrophages. *Parasitology Research*.

[35] Martelli AM, Faenza I, Billi AM, Falà F, Cocco L, Manzoli L (2003). Nuclear protein kinase C isoforms: key players in multiple cell functions?. *Histology and Histopathology*.

[36] Dekker LV, Parker PJ (1994). Protein kinase C—a question of specificity. *Trends in Biochemical Sciences*.

[37] Nishizuka Y (1988). The molecular heterogeneity of protein kinase C and its implications for cellular regulation. *Nature*.

[38] Newton AC (1997). Regulation of protein kinase C. *Current Opinion in Cell Biology*.

[39] Chakraborty R (1996). Oxygen-dependent Leishmanicidal activity of stimulated macrophages. *Molecular and Cellular Biochemistry*.

[40] Rodriguez-Pena A, Rozengurt E (1984). Disappearance of Ca^2+^-sensitive, phospholipid-dependent protein kinase activity in phorbol ester-treated 3T3 cells. *Biochemical and Biophysical Research Communications*.

[41] Moore KJ, Labrecque S, Matlashewski G (1993). Alteration of *Leishmania donovani* infection levels by selective impairment of macrophage signal transduction. *Journal of Immunology*.

[42] Descoteaux A, Turco SJ, Sacks DL, Matlashewski G (1991). *Leishmania donovani* lipophosphoglycan selectively inhibits signal transduction in macrophages. *Journal of Immunology*.

[43] Olivier M, Brownsey RW, Reiner NE (1992). Defective stimulus-response coupling in human monocytes infected with *Leishmania donovani* is associated with altered activation and translocation of protein kinase C. *Proceedings of the National Academy of Sciences of the United States of America*.

[44] McNeely TB, Rosen G, Londner MV, Turco SJ (1989). Inhibitory effects on protein kinase C activity by lipophosphoglycan fragments and glycosylphosphatidylinositol antigens of the protozoan parasite *Leishmania*. *Biochemical Journal*.

[45] Descoteaux A, Matlashewski G, Turco SJ (1992). Inhibition of macrophage protein kinase C-mediated protein phosphorylation by *Leishmania donovani* lipophosphoglycan. *Journal of Immunology*.

[46] Giorgione JR, Turco SJ, Epand RM (1996). Transbilayer inhibition of protein kinase C by the lipophosphoglycan from *Leishmania donovani*. *Proceedings of the National Academy of Sciences of the United States of America*.

[47] Chakraborty P, Ghosh D, Basu MK (1999). Macrophage protein kinase C: its role in modulating membrane microviscosity and superoxide in leishmanial infection. *Journal of Biochemistry*.

[48] Corradin S, Mauël J, Ransijn A, Stürzinger C, Vergères G (1999). Down-regulation of MARCKS-related protein (MRP) in macrophages infected with *Leishmania*. *Journal of Biological Chemistry*.

[49] Bhunia AK, Sarkar D, Das PK (1996). *Leishmania donovani* attachment stimulates PKC-mediated oxidative events in bone marrow-derived macrophages. *Journal of Eukaryotic Microbiology*.

[50] Pingel S, Wang ZE, Locksley RM (1998). Distribution of protein kinase C isoforms after infection of macrophages with *Leishmania major*. *Infection and immunity*.

[51] Desjardins M, Descoteaux A (1997). Inhibition of phagolysosomal biogenesis by the *Leishmania* lipophosphoglycan. *Journal of Experimental Medicine*.

[52] Holm A, Tejle K, Magnusson KE, Descoteaux A, Rasmusson B (2001). *Leishmania donovani* lipophosphoglycan causes periphagosomal actin accumulation: correlation with impaired translocation of PKC*α* and defective phagosoem maturation. *Cellular Microbiology*.

[53] Holm A, Tejle K, Gunnarsson T, Magnusson KE, Descoteaux A, Rasmusson B (2003). Role of protein kinase C *α* for uptake of unopsonized prey and phagosomal maturation in macrophages. *Biochemical and Biophysical Research Communications*.

[54] St-Denis A, Caouras V, Gervais F, Descoteaux A (1999). Role of protein kinase C-*α* in the control of infection by intracellular pathogens in macrophages. *Journal of Immunology*.

[55] Reiner NE, Malemud CJ (1984). Arachidonic acid metabolism in murine Leishmaniasis (*donovani*): ex-vivo evidence for increased cyclooxygenase and 5-lipoxygenase activity in spleen cells. *Cellular Immunology*.

[56] Farrell JP, Kirkpatrick CE (1987). Experimental cutaneous Leishmaniasis. II. A possible role for prostaglandins in exacerbation of disease in *Leishmania major*-infected BALB/c mice. *Journal of Immunology*.

[57] Matte C, Maion G, Mourad W, Olivier M (2001). *Leishmania donovani*-induced macrophages cyclooxygenase-2 and prostaglandin E synthesis. *Parasite Immunology*.

[58] Bhattacharyya S, Ghosh S, Sen P, Roy S, Majumdar S (2001). Selective impairment of protein kinase C isotypes in murine macrophage by *Leishmania donovani*. *Molecular and Cellular Biochemistry*.

[59] Bhattacharyya S, Ghosh S, Jhonson PL, Bhattacharya SK, Majumdar S (2001). Immunomodulatory role of interleukin-10 in visceral leishmaniasis: defective activation of protein kinase C-mediated signal transduction events. *Infection and Immunity*.

[60] Dey R, Majumder N, Bhattacharjee S (2007). *Leishmania donovani*-induced ceramide as the key mediator of Akt dephosphorylation in murine macrophages: role of protein kinase C*ζ* and phosphatase. *Infection and Immunity*.

[61] Dey R, Sarkar A, Majumder N (2005). Regulation of impaired protein kinase C signaling by chemokines in murine macrophages during visceral leishmaniasis. *Infection and Immunity*.

[62] Murray HW, Masur H, Keithly JS (1982). Cell-mediated immune response in experimental visceral leishmaniasis. I. Correlation between resistance to *Leishmania donovani* and lymphokine-generating capacity. *Journal of Immunology*.

[63] Belosevic M, Finbloom DS, Van der Meide PH, Slayter MV, Nacy CA (1989). Administration of monoclonal anti-IFN-*γ* antibodies in vivo abrogates natural resistance of C3H/HeN mice to infection with *Leishmania major*. *Journal of Immunology*.

[64] Igarashi KI, Garotta G, Ozmen L (1994). Interferon-*γ* induces tyrosine phosphorylation of interferon-*γ* receptor and regulated association of protein tyrosine kinases, Jak1 and Jak2, with its receptor. *Journal of Biological Chemistry*.

[65] Sakatsume M, Igarashi KI, Winestock KD, Garotta G, Larner AC, Finbloom DS (1995). The Jak kinases differentially associate with the *α* and *β* (accessory factor) chains of the interferon *γ* receptor to form a functional receptor unit capable of activating STAT transcription factors. *Journal of Biological Chemistry*.

[66] Lucas DM, Lokuta MA, McDowell MA, Doan JES, Paulnock DM (1998). Analysis of the IFN-*γ*-signaling pathway in macrophages at different stages of maturation. *Journal of Immunology*.

[67] Proudfoot L, Nikolaev AV, Feng GJ (1996). Regulation of the expression of nitric oxide synthase and leishmanicidal activity by glycoconjugates of *Leishmania* lipophosphoglycan in murine macrophages. *Proceedings of the National Academy of Sciences of the United States of America*.

[68] Reiner NE, Ng W, Ma T, McMaster WR (1988). Kinetics of *γ* interferon binding and induction of major histocompatibility complex class II mRNA in Leishmania-infected macrophages. *Proceedings of the National Academy of Sciences of the United States of America*.

[69] Nandan D, Reiner NE (1995). Attenuation of gamma interferon-induced tyrosine phosphorylation in mononuclear phagocytes infected with *Leishmania donovani*: selective inhibition of signaling through Janus kinases and Stat1. *Infection and Immunity*.

[70] Forget G, Gregory DJ, Olivier M (2005). Proteasome-mediated degradation of STAT1*α* following infection of macrophages with *Leishmania donovani*. *Journal of Biological Chemistry*.

[71] Ray M, Gam AA, Boykins RA, Kenney RT (2000). Inhibition of interferon-*γ* signaling by *Leishmania donovani*. *Journal of Infectious Diseases*.

[72] Bertholet S, Dickensheets HL, Sheikh F, Gam AA, Donnelly RP, Kenney RT (2003). *Leishmania donovani*-induced expression of suppressor of cytokine signaling 3 in human macrophages: a novel mechanism for intracellular parasite suppression of activation. *Infection and Immunity*.

[73] Matte C, Descoteaux A (2010). *Leishmania donovani* amastigotes impair gamma interferon-induced STAT1*α* nuclear translocation by blocking the interaction between STAT1*α* and importin-*α*5. *Infection and Immunity*.

[74] Feng GS, Pawson T (1994). Phosphotyrosine phosphatases with SH2 domains: regulators of signal transduction. *Trends in Genetics*.

[75] Kharitonenkov A, Chen Z, Sures I, Wang H, Schilling J, Ullrich A (1997). A family of proteins that inhibit signalling through tyrosine kinase receptors. *Nature*.

[76] Fischer EH, Charbonneau H, Tonks NK (1991). Protein tyrosine phosphatases: a diverse family of intracellular and transmembrane enzymes. *Science*.

[77] Charbonneau H, Tonks MK (1992). 1002 protein phosphatases?. *Annual Review of Cell Biology*.

[78] Marrero MB, Venema VJ, Ju H, Eaton DC, Venema RC (1998). Regulation of angiotensin II-induced JAK2 tyrosine phosphorylation: roles of SHP-1 and SHP-2. *American Journal of Physiology*.

[79] Banville D, Stocco R, Shen SH (1995). Human protein tyrosine phosphatase 1C (PTPN6) gene structure: alternate promoter usage and exon skipping generate multiple transcripts. *Genomics*.

[80] Yetter A, Uddin S, Krolewski JJ, Jiao H, Yi T, Platanias LC (1995). Association of the interferon-dependent tyrosine kinase Tyk-2 with the hematopoietic cell phosphatase. *Journal of Biological Chemistry*.

[81] David M, Chen HE, Goelz S, Larner AC, Neel BG (1995). Differential regulation of the alpha/beta interferon-stimulated Jak/Stat pathway by the SH2 domain-containing tyrosine phosphatase SHPTP1. *Molecular and Cellular Biology*.

[82] Ram PA, Waxman DJ (1997). Interaction of growth hormone-activated STATs with SH2-containing phosphotyrosine phosphatase SHP-1 and nuclear JAK2 tyrosine kinase. *Journal of Biological Chemistry*.

[83] Klingmuller U, Lorenz U, Cantley LC, Neel BG, Lodish HF (1995). Specific recruitment of SH-PTP1 to the erythropoietin receptor causes inactivation of JAK2 and termination of proliferative signals. *Cell*.

[84] Yi T, Cleveland JL, Ihle JN (1992). Protein tyrosine phosphatase containing SH2 domains: characterization, preferential expression in hematopoietic cells, and localization to human chromosome 12p12-p13. *Molecular and Cellular Biology*.

[85] Burshtyn DN, Yang W, Yi T, Long EO (1997). A novel phosphotyrosine motif with a critical amino acid at position 2 for the SH2 domain-mediated activation of the tyrosine phosphatase SHP-1. *Journal of Biological Chemistry*.

[86] Frearson JA, Alexander DR (1997). The role of phosphotyrosine phosphatases in haematopoietic cell signal transduction. *BioEssays*.

[87] Berg KL, Carlberg K, Rohrschneider LR, Siminovitch KA, Stanley ER (1998). The major SHP-1-binding, tyrosine-phosphorylated protein in macrophages is a member of the KIR/LIR family and an SHP-1 substrate. *Oncogene*.

[88] Olivier M, Romero-Gallo BJ, Matte C (1998). Modulation of interferon-*γ*/-induced macrophage activation by phosphotyrosine phosphatases inhibition: effect on murine leishmaniasis progression. *Journal of Biological Chemistry*.

[89] Nandan D, Reiner NE (2005). *Leishmania donovani* engages in regulatory interference by targeting macrophage protein tyrosine phosphatase SHP-1. *Clinical Immunology*.

[90] Nandan D, Tran T, Trinh E, Silverman JM, Lopez M (2007). Identification of leishmania fructose-1,6-bisphosphate aldolase as a novel activator of host macrophage Src homology 2 domain containing protein tyrosine phosphatase SHP-1. *Biochemical and Biophysical Research Communications*.

[91] Abu-Dayyeh I, Shio MT, Sato S, Akira S, Cousineau B, Olivier M (2008). *Leishmania*-induced IRAK-1 inactivation is mediated by SHP-1 interacting with an evolutionarily conserved KTIM motif. *PLoS Neglected Tropical Diseases*.

